# Minimally designed thermo-magnetic dual responsive soft robots for complex applications†

**DOI:** 10.1039/d3tb02839a

**Published:** 2024-03-28

**Authors:** Clio Siebenmorgen, Chen Wang, Laurens Bosscher Navarro, Daniele Parisi, Sarthak Misra, Venkatasubramanian Kalpathy Venkiteswaran, Patrick van Rijn

**Affiliations:** a University of Groningen, University Medical Center Groningen, Biomaterials & Biomedical Technology, Deusinglaan 1 Groningen 9713 AV The Netherlands p.van.rijn@umcg.nl; b University of Groningen, Faculty of Science and Engineering, Product Technology – Engineering and Technology Institute Groningen, Nijenborgh 4 9747 AG Groningen The Netherlands; c Surgical Robotics Laboratory, Department of Biomechanical Engineering University of Twente, Drienerlolaan 5 7522 NB Enschede The Netherlands v.kalpathyvenkiteswaran@utwente.nl

## Abstract

The fabrication of thermo-magnetic dual-responsive soft robots often requires intricate designs to implement complex locomotion patterns and utilize the implemented responsive behaviors. This work demonstrates a minimally designed soft robot based on poly-*N*-isopropylacrylamide (pNIPAM) and ferromagnetic particles, showcasing excellent control over both thermo- and magnetic responses. Free radical polymerization enables the magnetic particles to be entrapped homogeneously within the polymeric network. The integration of magnetic shape programming and temperature response allows the robot to perform various tasks including shaping, locomotion, pick-and-place, and release maneuvers of objects using independent triggers. The robot can be immobilized in a gripping state through magnetic actuation, and a subsequent increase in temperature transitions the robot from a swollen to a collapsed state. The temperature switch enables the robot to maintain a secured configuration while executing other movements *via* magnetic actuation. This approach offers a straightforward yet effective solution for achieving full control over both stimuli in dual-responsive soft robotics.

## Abstract 2

The minimally designed robot (THANOS) showcases excellent control over both thermo- and magnetic responses. The robot performs various tasks including shaping, locomotion, pick-and-place, and release maneuvers of objects using independent triggers.

## Graphical abstract

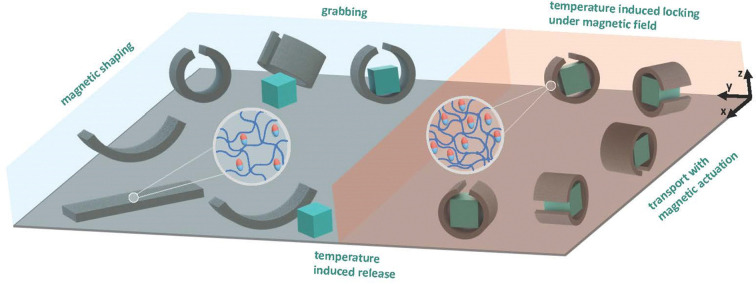

## 1 Introduction

For many engineering inventions, nature has served as a profound source of inspiration. In the field of robotics, insects have been an inspiration for the design of agile and adaptable robots, which can navigate through complex terrains.^1,2^ Marine organisms such as cephalopods have influenced the research progress in soft robotics, as they show complex locomotion such as crawling, and jet propulsion.^3,4^ Furthermore, cephalopods can undergo significant changes in body shape through muscular contractions to access confined spaces. This capability results from the soft properties and has led to biomimetic systems in the field of soft robotics.

Soft robots are made of flexible materials such as elastomers, gels, and polymers and contain domains that react to external stimuli, such as light, temperature, pH, acoustic waves, and electric and magnetic fields.^5–11^ Among these stimuli, magnetic actuation stands out as a particularly promising method, offering various advantages, such as non-invasiveness, remote control capability, suitability for small-scale applications, and safety for medical use.^12–14^ Using magnetic actuation in soft robotics is particularly useful in the context of biomedical applications, including the utilization of surgical robots.^14–19^ Numerous examples of magnetically activated robots have been reported previously, where the majority of robots are based on polymeric materials, such as silicon elastomers, in which magnetic nanoparticles have been incorporated.^20–29^ These robots show high flexibility, facile synthesis, and can be synthesized to be biocompatible. However, they rely solely on magnetic actuation where each soft robot is limited to actuation modes in a soft state, limiting their potential functionality. These robots cannot be altered or switched to different modes of actuation without modifications to their design or operating principles.

Creating dual-responsive soft robots allows the incorporation of combined functionalities by integrating multiple responsive mechanisms. By employing temperature-responsive polymeric networks with incorporated magnetic particles, a dual responsive thermo-magnetic system can be realized. Poly(*N*-isopropylacrylamide) (pNIPAM)-based hydrogels are a popular choice of material, as this polymer undergoes phase-transition close to body temperature at around 32 °C.^30,31^ It exhibits phase transition from a hydrophilic to a hydrophobic state when the temperature increases above its volume phase transition temperature (VPTT). Systems have been developed that enable either grabbing, transport, or different forms of locomotion. However, they require complex designs and the use of several polymeric carrier systems to provide sufficient strength or induce the desired function. Particularly, the use of the magnetic field for different functions in one system remains challenging. Therefore, a design without any specific added complexity will potentially enhance its applicability as well as its down-scaling as the complexity will require small features for *in vivo* applications. Previously, pNIPAM-based thermo-responsive soft robots have been studied.^32^ Breger *et al.* synthesized a double layer polymeric gripper, in which the first layer was based on the pNIPAM-*co*-acrylic acid swelling hydrogel and the second layer consisted of non-swelling propylene furmarate polymers.^33^ They further incorporated Fe_2_O_4_ nanoparticles for a dual thermo-magnetic response. Due to the phase transition of the thermo-responsive hydrogel, these soft robots demonstrated the capability of self-folding gripping motions when increasing the temperature above the VPTT. However, a complex design of a star shaped double layer polymeric sheet is necessary to achieve sufficient strength during gripping motion. Furthermore, variations in motion in response to the temperature increase pose a challenge due to the intricate nature of the design. Du *et al.* used a pNIPAM polymer-based millirobot with a magnetized head group by incorporating NdFeB particles and a non-functionalized pNIPAM tail group.^34^ This millirobot showed multimodal locomotion, such as crawling, helical propelling, and rolling. However, all locomotions can only be achieved when applying a magnetic field.

**Fig. 1:**
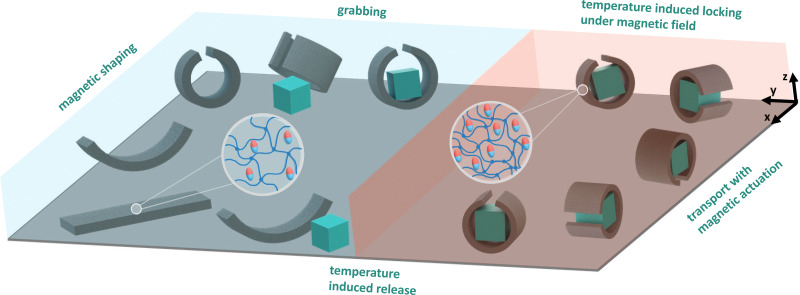
Schematic illustration depicting THANOS and its responsive behavior to thermal and magnetic stimuli, enabling pick-and-place maneuvers of objects. When heated, THANOS expels water and is shape locked. Upon cooling, THANOS absorbs water and becomes flexible for magnetic shaping.

The work presented here takes a different approach towards dual responsive **th**ermo-m**a**g**n**etic s**o**ft robotic**s** (THANOS) based on pNIPAM and ferromagnetic particles. The design offers a substantially simplified synthesis method achieved through one-step temperature-induced free radical polymerization without the need for depositing different polymer classes with spatial control. The key novelty of THANOS lies in the combination of simplicity in design with the integration of magnetic shape programming and temperature response to ensure shape-locking (Fig. 1). When exposed to a magnetic field, the robot can perform various complex locomotions, such as rolling, undulating, and inchworm-like motion. Simultaneously, the robot can be locked in the desired shape by increasing the temperature above the VPTT without the need for magnetic actuation. This capability empowers the robot to execute dual functions. Initially, the robot can be immobilized in a gripping (or any other) state by magnetic actuation. By raising the temperature and then deactivating the magnetic actuation, the robot can be fixed in the desired shape. Following this, the robot can execute secondary movements through magnetic actuation while remaining in the secured configuration. This control mechanism could be particularly valuable in application such as minimally invasive surgery, enabling the robot to perform dual tasks through independent stimuli that would otherwise be challenging.

## 2 Materials and methods

### 2.1 Chemicals and materials

*N*-Isopropylacrylamide (>98%, NIPAM) was purchased from Tokyo Chemical Industry (TCI), Belgium. *N*,*N*′-methylenebis(acrylamide) (99%, BIS), potassium persulfate (99%, KPS), and poly(dimethylsiloxane) (PDMS) were purchased from Sigma-Aldrich, The Netherlands. *N*,*N*,*N*,*N*′-tetramethylethylenediamine (TEMED) was purchased from Bio-Rad Laboratories, United States of America. MQFP^TM^-16-7-11277 5 μm sized PrFeB particles were obtained from Magnequench GmbH, Germany. All chemicals were used as received without any further purification. Ultra-pure water (18.2 MΩ, arium 611 DI water purification system; Sartorius AG, Germany) was used for all experiments.

### 2.2 Preparation of molds

The fabrication of PDMS molds for the preparation of pNIPAM sheets involves a two-step molding process. Initially, a negative mold featuring a 30 × 30 × 0.5 mm boss is created through laser cutting of acrylic (poly-methyl methacrylate) (PMMA). Subsequently, PDMS is poured into the mold and subjected to curing at 70 °C for a duration of 4 hours. Following the curing process, the PDMS molds, characterized by a 30 × 30 × 0.5 mm groove, are obtained by peeling off from the PMMA negative mold.

To achieve customized shapes in the maternal sheets, molds of the desired configurations are laser-cut and equipped with a thin wall serving the purpose of a cutting implement. This approach allows for precision in shaping the maternal sheets, ensuring the attainment of specific geometries to meet the unique requirements of the study.

### 2.3 Synthesis of pNIPAM sheets

For the synthesis of THANOS sheets, 700 mg of MQFP^TM^-16-7-11277 particles were dispersed in 10 mL of ultra-pure water in a 20 mL vial and were subsequently tip sonicated for 30 s using a Vibra cell (Sonic and Materials Inc., Danbury, Connectitcut, USA) with a duty cycle of 60% set at an output control of 5. To this suspension, 566 mg of NIPAM (0.5 M) and 154 mg of BIS (0.1 M) were added and subsequently degassed with N_2_ for 15 min. In a 2 mL vial, 4 mg KPS was dissolved in 1 mL of ultra-pure water and subsequently degassed with N_2_ for 15 min. The solutions were transferred to a nitrogen atmosphere and were stored in an ice bath to extend the duration of the free radical polymerization reaction to approximately 5 seconds. Prior to crosslinking, 700 μL of the monomer-particle suspension was transferred to a 1.5 mL Eppendorf tube, in which 10 μL of KPS initiator was added. With continuous agitation, the mixture was then poured into the previously prepared molds and covered with a round PMMA cover slip with a diameter of 60 mm. The soft robot was subsequently washed with ultra-pure water and stored in ultra-pure water at room temperature.

The preparation of pNIPAM@Control sheets followed the same procedure. However, MQFP^TM^-16-7-11277 particles have not been added to the monomer solution and therefore it was not subjected to tip sonication. Furthermore, additional 10 μL of TEMED have been added simultaneously with 10 μL of KPS to the mixture to initiate polymerization.

### 2.4 Magnetization

Following the synthesis process, the sheets undergo a transformation process facilitated by placement into a specialized non-magnetic fixture designed for shaping purposes. The sheet, in conjunction with the fixture, is subsequently subjected to a 2 T magnetic field. The magnetic field is generated utilizing an impulse magnetizer (ASC Model IM-10-30, ASC Scientific, USA) to achieve the targeted magnetization profile as per the experimental requirements.

### 2.5 Scanning electron microscopy sample preparation

To image the cross-section of the sheets, they were frozen with N_2(l)_, broken in half and subsequently freeze dried for 24 h using a Labogene Scanvac Coolsafe freeze dryer at a condenser temperature of −110 °C. Double 90° angled Zeiss short pin stubs with a diameter of 25.4 mm were covered using carbon-based adhesive disks. The cross-section of the pNIPAM sheet was then placed on the double 90° short pin stubs and subsequently sputter coated with 5 nm Cr with a working distance of 50 mm, a stab height of 15 mm, and a tilt stage angle of −10°. The images were taken using an Atlas Zeiss Supra 55 STEM scanning electron microscope with a field emission gun running at 3 kV. MQFP^TM^-16-7-11277 particles have been imaged using a stub with a diameter of 9 mm, covered with a carbon-based adhesive disk, and were subsequently sputter coated with 5 nm Cr with a working distance of 15 mm, and a stab height of 15 mm. The images were taken using the same scanning electron microscope, but due to charging of the sample, the field emission gun was running at 2 kV.

### 2.6 Volume phase transition temperature

To test the temperature response of THANOS and pNIPAM@Control sheets, the degree of surface area reduction of the sheets due to gradual temperature changes (1 °C min^−1^) was monitored. For this purpose, the sheets were placed on a mm grid inside a water bath on top of a heating plate with temperature control. The sheets were then imaged using a standard and infrared camera. The infrared camera allowed for a double control of the actual temperature of the sheets. Subsequently, the percentage of the surface area was plotted against the temperature.

### 2.7 Time-dependent shrinking

To investigate the time-dependant shrinking of THANOS and pNIPAM@Control, both sheets were transferred from a water bath at room temperature to a heated water bath at 50 °C while monitoring the reduction of the surface area. For this purpose, the sheets with a dimension of 15 × 5 mm were placed on a mm grid inside a water bath at 50 °C on top of a heating plate with temperature control. The sheets were then imaged using a standard camera and the measurement was repeated five times. The percentage of the surface area was subsequently plotted against the time.

### 2.8 Rheological characterization

Rheological experiments were performed in a Discovery Hybrid Rheometer (HR-2) obtained from TA Instruments (United States). 25 mm diameter parallel plates were used for all the rheological tests. The samples were loaded into the rheometer, and dynamic strain sweeps were executed at 100 rad s^−1^ to determine a strain within the linear viscoelastic (LVE) regime. Consequently, frequency sweeps were performed over a range of frequencies varying from 100 to 0.1 rad s^−1^, and oscillatory strain amplitude between 1 and 10%. All the measurements were carried out either at room temperature or at 50 °C. The temperature control system consisted of an electric element coupled with a convection oven, fed with nitrogen gas at a very low flow rate (<1 L min^−1^).

## 3 Results

### 3.1 Fabrication of pNIPAM-based soft robotic material

pNIPAM@Control and THANOS sheets followed a similar free radical polymerization approach. In contrast, pNIPAM@Control required the addition of TEMED and KPS to initiate polymerization, whereas THANOS robotic sheets could be initiated solely with KPS. KPS will readily homolytically cleave upon increasing the temperature, even at room temperature, and induce free radical polymerization. However, before initiating the free radical polymerization of THANOS sheets, the aqueous suspension containing solely PrFeB particles was tip sonicated using low frequency ultrasonic waves. Subsequently, NIPAM and BIS were added to the suspension. Without this additional step, free radical polymerization did not occur.

**Fig. 2:**
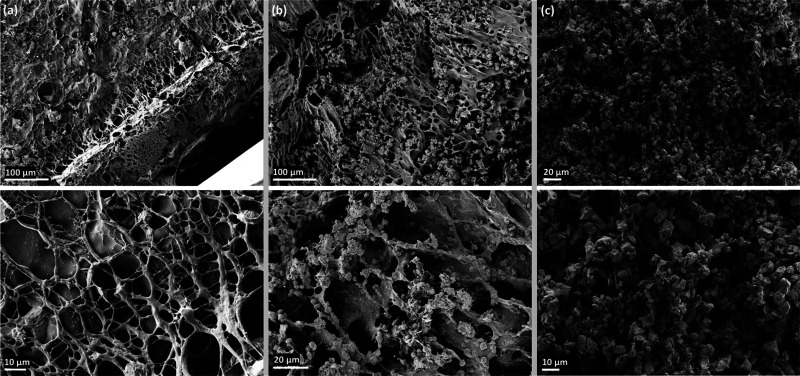
SEM images depict (a) the cross-section of the pNIPAM@Control sheet after freeze drying, (b) the cross-section of THANOS after freeze drying, showcasing a uniform dispersion of ferromagnetic particles within the hydrogel, and (c) ferromagnetic MQFP^TM^-16-7-11277 particles observed as a powder without being incorporated into a polymeric matrix.

Sonication of aqueous solutions leads to the cavitation of present microbubbles, which ultimately results in so called ‘hot spots’. These localized hot spots give rise to extreme conditions, with temperatures above 5000 K and pressures exceeding 1000 atm.^35^ There are two possible explanations for the initiation of the polymerization of THANOS robotic sheets without the addition of TEMED: (1) the conditions created by hot-spots could cause defects on the surface of the MQFP^TM^ particles, which could ultimately enhance the surface reactivity. (2) Ultrasonication of the suspension could lead to the generation of reactive species. These reactive species could act as initiation sites when in contact with KPS. Sonication-induced radical formation of the monomer-particle suspension allowed a complete polymerization of the sheets within seconds, while the reaction of the control group took several minutes. The extremely short reaction time is crucial for the synthesis of THANOS robotic sheets since the MQFP^TM^ particles would otherwise sediment. Despite minimizing sedimentation by the reduced reaction time and suspending the reaction mixture before crosslinking, the upper section of the robot exhibited slight discoloration. This suggests that particles with a slightly lower concentration reside within the top of the polymeric matrix. To obtain a better understanding of the macroscopic features of the polymeric sheets and the subsequent incorporation of magnetic particles within the THANOS robot, SEM images of the cross-section of both sheets have been taken. Fig. 2 shows SEM images of (a) the cross section of the previously freeze-dried NIPAM@Control sheet, (b) the cross section of the previously freeze-dried THANOS sheet and (c) ferromagnetic particles MQFP^TM^-16-7-11277, which are not incorporated inside a polymeric matrix. In Fig. 2(b), the successful and overall uniform integration of ferromagnetic particles into the polymer matrix of the pNIPAM sheets is demonstrated. As described previously, the reaction rate of free radical polymerization of THANOS sheets influences the distribution of particles. An increase in the reaction rate is crucial, as high density MQFP^TM^ particles would otherwise sediment especially, when relying on a different initiation system.

### 3.2 Rheological characterization of pNIPAM sheets

Rheological experiments were performed to evaluate how the presence of magnetic particles and temperature variations affect the rheological properties of the polymeric sheets. Fig. S3 (ESI†) shows the complex viscosity and complex modulus of THANOS depending on the angular frequency at 25 °C and 50 °C, while Fig. S4 (ESI†) displays the rheological results of pNIPAM@Control. Increasing the temperature from 25 °C to 50 °C leads to an increase in complex viscosity and complex modulus in both cases, for THANOS and for pNIPAM@Control. Furthermore, the incorporation of magnetic particles within the polymeric network leads to an increase in complex viscosity at 25 °C at an angular frequency of 0.1 rad s^−1^ by a factor of 10, while the complex modulus increases by a factor of 1000. Increasing the temperature above the VPTT to 50 °C increases the complex viscosity and complex modulus in both THANOS and pNIPAM@Control. THANOS shows increased values for the complex viscosity and complex modulus at an angular frequency of 0.1 rad s^−1^ by a factor of 100 when compared to pNIPAM@Control at 50 °C. There are two phenomena that can explain the difference in the complex modulus and complex viscosity of THANOS and pNIPAM@Control. First, the integration of rigid ferromagnetic particles into the dual-responsive sheet can increase the overall modulus. The incorporation of magnetic particles within the polymeric sheet reduces the overall flexibility due to a higher resistance of deformation, ultimately leading to a stiffer material. The reinforcement effect of inorganic nanoparticles for hydrogels has been reported previously but still offers enough flexibility to take on magnetically induced shapes and various modes of induced locomotion.^36^ Secondly, the reaction time, and thus, the kinetics, of the free radical polymerization of pNIPAM@Control compared to THANOS sheets differs significantly. This effect may alter the crosslinking density, which will ultimately influence the resulting rheological properties of the sheet.

### 3.3 Thermo-responsive behaviour of pNIPAM sheets

As described previously, pNIPAM networks are thermo-responsive, in which the VPTT refers to the temperature under which the polymeric network switches from a swollen to a collapsed state by absorbing or repelling water from the polymeric network.^30^ Below the VPTT, pNIPAM shows hydrophillic characteristics and undergoes hydrogen bonding with water molecules *via* the amide groups of the polymer and is therefore in the swollen/hydrated state. Typically, the VPTT of pNIPAM hydrogels is around 32 °C.^37^ At this temperature, the polymer undergoes conformational change and starts to collapse under which the hydrogen bonds to water weaken. The polymeric network becomes more hydrophobic. However, several factors, such as the crosslinking density, presence of salts and ionic species in the solution, and the polarity of copolymers influence the VPTT.^38–43^ Typically, copolymerization with more hydrophobic comonomers will shift the VPTT towards higher temperatures, whereas increased hydrophilicity of comonomers decreases the VPTT.

**Fig. 3:**
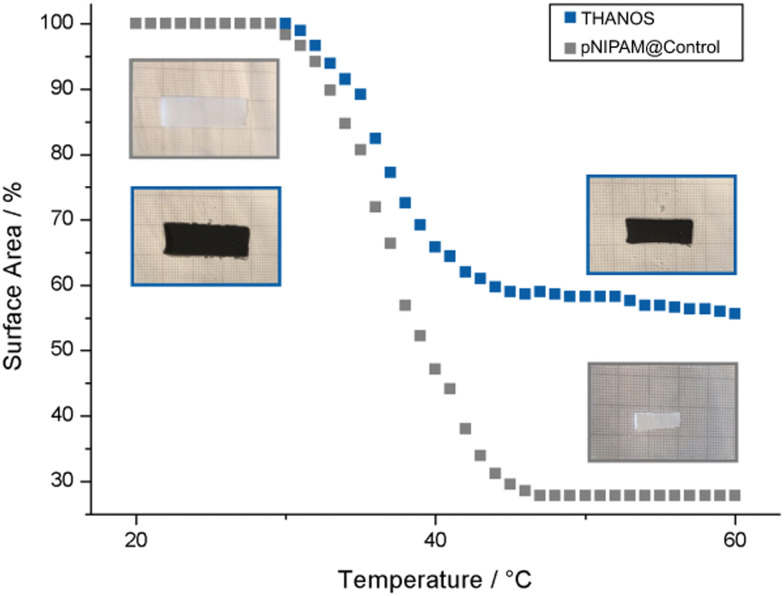
Characterization of the VPTT by plotting the percentage of the surface area based on the swollen state of pNIPAM@Control (grey) and THANOS (blue) against the temperature.

To characterize the temperature responsive behavior of pNIPAM-based sheets, the surface area of each sheet has been plotted against the temperature. For a better visualization of the reduction in surface area upon elevating the temperature, sheets with a dimension of 30 × 10 mm were used. The surface area is plotted against the temperature, in which 100% on the *y*-axis represents the surface area of the sheet in the swollen state at room temperature. Fig. 3 displays the thermo-responsive characteristics of THANOS and pNIPAM@Control sheets at temperatures between 24 °C and 60 °C by gradually increasing the temperature of the water bath at a rate of 1 °C min^−1^. The VPTT of THANOS and pNIPAM@Control is at 37 °C. As mentioned earlier, pNIPAM typically exhibits a VPTT of 32 °C. The variance in the VPTT observed in both pNIPAM@Control and THANOS can be attributed to the overall chemical composition of the polymeric sheets.

Furthermore, THANOS has a maximum surface area shrinkage of 44%, while pNIPAM@Control shows a 72% reduction in surface area upon elevated temperatures. The significant reduction in the shrinkage capability of THANOS can be explained by the integration of rigid ferromagnetic particles, ultimately, limiting the flexibility of the polymeric chains.

Additionally, the time-dependant shrinking capability of THANOS and pNIPAM@Control at 50 °C was examined. At 50 °C, a fully collapsed state of both sheets is expected. For this purpose, each sheet was placed from a water bath at room temperature to a heated water bath at 50 °C and the reduction of the surface area was subsequently plotted against the time (Fig. S2, ESI†). The shrinking capability is fast and the maximum reduction in surface is after 45 s for both pNIPAM@Control and the THANOS sheet. The overall reduction shows only a slight deviation in the maximum shrinking capability when compared to the results obtained by gradually increasing the surrounding temperature (Fig. 3). Therefore, it can be concluded that an instantaneous increase in temperature leads to similar shrinking results.

### 3.4 Magnetic-responsive behaviour of the robot

The mechanical behaviour of the robot under an external magnetic field is studied using simulations and experiments. Three magnetic profiles are investigated: S, L and C. A setup consisting of six electromagnetic coils arranged in a Helmholtz configuration with a workspace of 12 × 12 × 12 cm is utilized for magnetic actuation (see Fig. S5, ESI†). This setup can generate a uniform magnetic field of up to 50 mT in any given direction. Two cameras are employed for observing and recording the top and side views of the experiments.

**Fig. 4:**
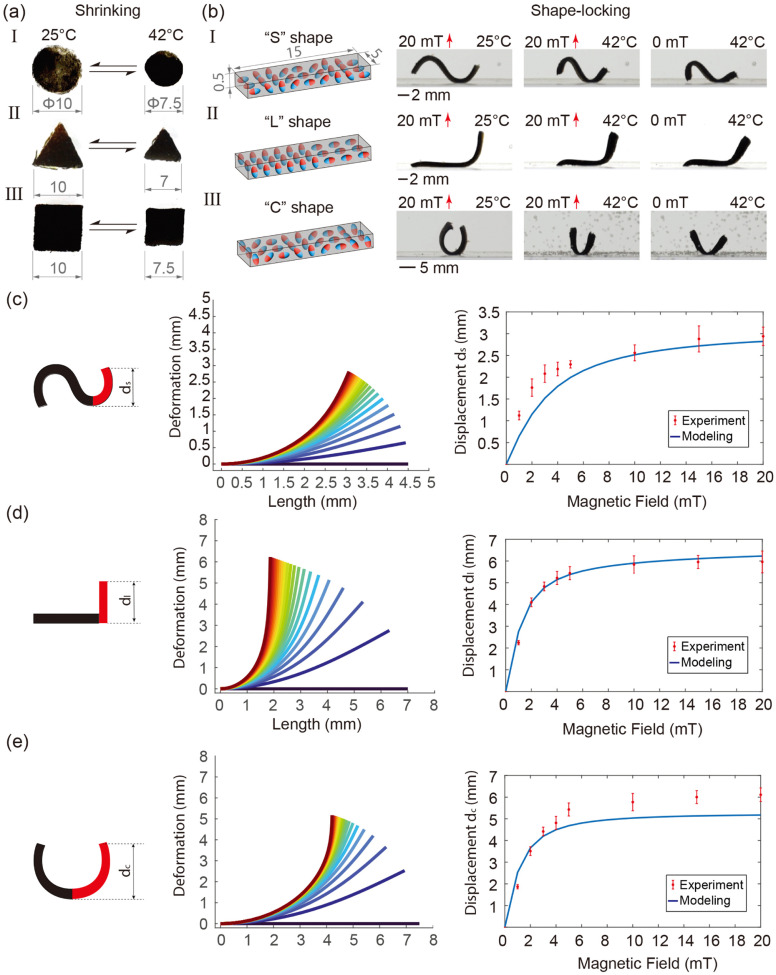
Thermal-shrinking and shape-locking features of the dual responsive sheets. (a) Reversible thermal shrinking of different shapes, namely circular (1), triangular (2) and rectangular (3). The original dimensions are measured at room temperature (25 °C), while the shrunken dimensions are measured at the completed shrinking point at 42 °C as characterized. Dimensions are in mm. (b) Magnetic programming and shape-locking property of the three typical geometry shapes are shown. The magnetization profile of each shape is indicated in the first column of the figures. The red solid arrows indicate the direction of the applied magnetic field. Simulation results of deformation of the (c) “S”, (d) “L” and (e) “C” shaped robot. The deformation of the right quarter of the robot under increased magnetic field strength from 0 mT to 20 mT is shown in the middle graph. Both experiment and simulation results of the displacement of the right tip are shown in the right graph.

The Cosserat rod theory-based model is utilized to simulate the deformation of the robot. The detailed modelling method is described in Section A, ESI.† A comparison between experiments and simulation results on the deformation of the three magnetic profiles is shown in Fig. 4(c–e). These results indicate that the deformation of the robot increases rapidly at lower magnetic fields and plateaus out around 10 mT. This phenomenon arises due to the decrease in magnetic torque acting on the robot body as the magnetic particles within the robot body align with the direction of the magnetic field. More simulation and experiment results are found in Fig. S6–S10 (ESI†).

It is important to note that the shrinkage of the robot results in changes not only in its volume but also in the relative concentration of magnetic powder within the robot. As a result, the magnetic moment which actuates the robot also changes. Fig. S8 (ESI†) illustrates the impact of powder concentration on the deformation of the robot under magnetic field strengths ranging from 0 mT to 10 mT. This demonstrates that displacement increases rapidly at low powder concentrations and gradually at higher concentrations. Additionally, the shrinkage of the robot results in an increase in stiffness. The effect of robot stiffness on displacement under magnetic fields ranging from 0 mT to 20 mT is studied and depicted in Fig. S9 (ESI†). It is evident that higher stiffness leads to a reduction in robot deformation.

### 3.5 Thermo-magnetic dual responsive properties

The pNIPAM sheet mixed with magnetic particles responds to both environment temperature and external magnetic field. The design flexibility of the sheet enables the creation of various planar shapes through mold design. In order to demonstrate the isotropic shrinkage, three different shapes (circle, triangle, square) are tested as shown in Fig. 4(a) (please refer to supplementary Video, ESI†). A setup is designed to control the temperature of the water in a tank. Temperature adjustments are achieved by utilizing a resistance heater for heating and pumping of cold water for cooling. In accordance with the VPTT results, the water temperature is calibrated to reach up to 50 °C. The initial dimensions of the planar sheet are measured at room temperature (25 °C), while the shrunken size is determined at the completion of the shrinking process (42 °C). As illustrated in Fig. 4(a), the sheets show isotropic shrinkage as they retain their original shapes in the collapsed state, which further demonstrates a homogeneous distribution of magnetic particles inside the polymeric matrix.

By programming the magnetization profiles, the sheet can undergo deformation into predefined shapes when subjected to an external magnetic field. Simultaneously, the stiffness of the sheet exhibits an upward trend with increasing temperature. Consequently, the shape induced by the external magnetic field can be locked in place upon reaching the completed shrinking point, subsequent to the removal of the magnetic field. Fig. 4(b) (please refer to supplementary Video, ESI†) showcases the programmable magnetization and shape-locking characteristics of the sheet, featuring the letters “S”, “L”, and “C”. Initially, a constant magnetic field is applied in a fixed direction, as indicated by the red arrows in the figure. The magnetic particles within the sheets align with the magnetic field direction, generating a magnetic torque on the sheet and inducing the intended deformations. During this process, the water is heated while maintaining the magnetic field to preserve the formed shape. We determined the VPTT of THANOS and pNIPAM@Control to be 37 °C, which represents the state where the gel is between fully swollen and fully collapsed. The fully shrunken state is needed for locking the shape which is reached at 42 °C (see Fig. 3). Therefore, the ultimate shapes are conclusively locked at 42 °C, coinciding with the deactivation of the magnetic field. While here external heating is used to induce the collapse of the network, it is known that hyperthermia approaches (heating by alternating magnetic fields) are also possible with pNIPAM.^44^

### 3.6 Multimodal locomotion of the sheet-shaped robot

**Fig. 5:**
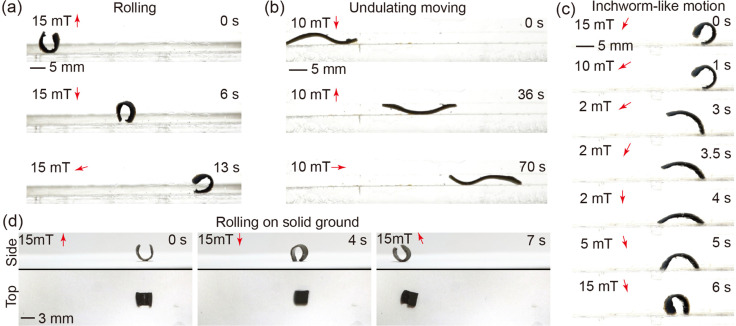
Demonstration of multimodal locomotion of the sheet-shaped robot. (a) The robot exhibits the ability to roll underwater using a rotating magnetic field at 15 mT and 0.2 Hz. (b) The robot executes undulating movements within a channel with a height of 3 mm, achieved under a rotating magnetic field at 10 mT and 0.2 Hz. (c) The robot's capacity to perform inchworm-like motion underwater by applying a specialized swing magnetic field as shown. (d) The robot, at a semi-dried status, showcases the capability to roll on solid ground outside water, propelled by a rotating magnetic field at 15 mT and 0.2 Hz.

The locomotion capabilities of the minimally designed (sheet structure) robot are demonstrated across various motion patterns and environmental settings (please refer to supplementary Video, ESI†). In particular, the “C” shaped magnetized sheet is chosen for this study to showcase the robot's motion abilities. Under the influence of a rotating magnetic field, the robot adeptly executes underwater rolling, as depicted in Fig. 5(a). The robot further demonstrates its capacity to move within a channel through undulating wave motions induced by a rotating magnetic field, as illustrated in Fig. 5(b). Moreover, the robot exhibits the ability to navigate underwater in an inchworm-style gait by employing a swing magnetic field, as evidenced in Fig. 5(c). Notably, the robot can also transition to terrestrial locomotion by rolling on solid ground outside an aqueous environment. This transition is achieved by eliminating water from the robot's inner body to increase the magnetic particle concentration and removing residual water on the body surface to reduce the adhesion force on the solid ground, as depicted in Fig. 5(d). The dehydration process is executed using a hot wind gun. The amphibious locomotion ability broadened the potential application area of the robot.

To compare the aforementioned motion patterns, the displacement per motion cycle is experimentally calculated for each motion pattern under magnetic fields ranging from 0 mT to 20 mT and magnetic frequencies ranging from 0.1 Hz to 1 Hz. The comparison results (shown in Fig. S10, ESI†) reveal that rolling motion exhibits the highest displacement per actuation cycle, whereas undulating motion demonstrates the lowest. Notably, the speed of rolling on solid ground is lower than that underwater, primarily due to changes in the robot's dimensions caused by the absence of water within its body.

### 3.7 Multifunction of the robot

**Fig. 6:**
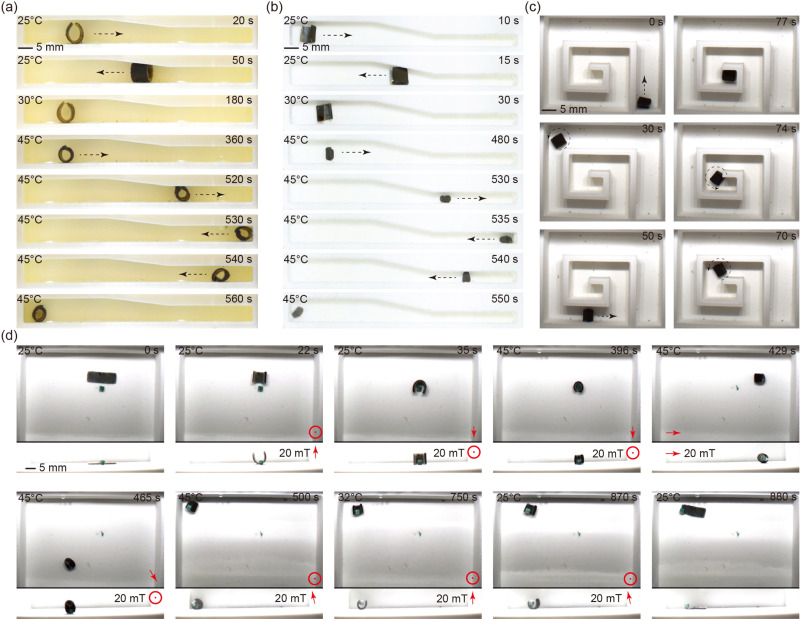
Demonstrations of robot maneuverability and functions. (a) and (b) The robot capability of transition from a spacious channel to a narrower channel by changing the size of the robot. (c) The robot can be controlled to navigate in a maze-like structure. (d) The robot is able to pick up, transport, and release an object (3 × 3 × 3 mm cube) to a target place by the control of magnetic field and temperature.

Based on the properties of thermal shrinking, shape locking and magnetic actuation, the robot exhibits a unique capability to reach target locations and perform specialized functions that pose challenges for other magnetic soft robots. As shown in Fig. 6(a) (please refer to supplementary Video, ESI†), the robot, after undergoing shrinking, successfully navigates through a narrow channel (4.5 mm in height), overcoming its original diameter of 6 mm at room temperature. Similarly, by elevating the water temperature, the robot adeptly maneuvers through a narrow channel (3 mm in width), which is narrower than its initial width of 5 mm, as illustrated in Fig. 6(b) (please refer to supplementary Video, ESI†). The steering of the robot is accomplished by adjusting the tilt angle of the rotating magnetic field plane (for detailed control methodology, refer to our previous work).^45^ This maneuverability is demonstrated as the robot navigates a designed maze, as shown in Fig. 6(c) (please refer to supplementary Video, ESI†). The pick-and-place function is exemplified in the subsequent experiment depicted in Fig. 6(d) (please refer to supplementary Video, ESI†). Initially, the robot rolls up and flips to cover an object, specifically a 3 × 3 × 3 mm 3D printed poly(lactic acid) (PLA) cube. Upon increasing the temperature to 45 °C, while maintaining the magnetic field, the robot undergoes full shrinkage, successfully grasping the object. The shrunken robot, along with the object, is then flipped back and rolled to the target location under magnetic field control while maintaining the temperature at 45 °C. Upon reaching the target location, the magnetic field is deactivated, and the water temperature is lowered by circulating cold water into the tank and removing the heated water from it. Subsequently, the robot reverts to its original size and soft state, leading to the release of the object at the target location.

## 4 Discussion

The experimental results support the characterization results. By increasing the temperature of the environment, we observed the changes on the size of the sheets. It is worth noting that THANOS robots undergo isotropic shrinkage, ultimately validating the homogeneous distribution of particles within the polymeric matrix as shown in Fig. 4(a). Enhancing the molding process can result in improved precision and refinement of the edges and shapes of the sheets. Also, more complicated planar shapes can be made based on the fabrication method presented in this article. Furthermore, exploring the potential of employing advanced manufacturing techniques such as photolithography or 3D printing is intriguing, as it could enable the downsizing of the robot, making it more accessible to confined and enclosed spaces for potential medical applications that are otherwise difficult to reach.^33,46–48^ However, validation is essential; for instance, rheological modifiers are sometimes necessary for 3D printing, which may ultimately impact the resulting mechanical properties of the robot but there are alternatives available that offer the same control and network formations as depicted in the THANOS system.^49^

The locomotive capabilities of the robot in the swollen state are demonstrated through actuation using a magnetic field. This work presents various motion patterns, including rolling, undulating, and inchworm-like motion. The frequency of the rotating magnetic field utilized for rolling and undulating movements is set at 0.2 Hz, with the possibility of enhancing the movement speed by adjusting the frequency in accordance with specific requirements.

Typically, hydrogel-based magnetic soft robots can only be actuated underwater due to the buoyancy provided, which overcomes body weight and compensates for the low actuation force/torque. It is noteworthy that in the collapsed state, the THANOS robot demonstrates the ability to move on solid ground outside water, a phenomenon not previously observed. This capability arises from the temperature responsive behavior of the hydrogel, in which an increase in temperature weakens the hydrogen bonding of the amide groups of pNIPAM with water, ultimately resulting in an increase in hydrophobic interactions, and thus, leading to repulsion of water. This phenomenon increases the ratio of magnetic particles within the polymeric network.^50^ Consequently, the magnetic torque acting on the robot body can surpass the body weight in the air. This unique feature holds significant implications for potential future applications, particularly in medical scenarios, such as the gastrointestinal tract, where both dry and wet conditions exist. The amphibious locomotion ability of the robot shows great potential to access challenging locations that may pose difficulties for other robotic systems. Future studies with a clear biomedical application to which THANOS can be tailored in terms of mechanical/(bio)chemical/responsive properties need to confirm the locomotive capabilities and function of THANOS in environments that are relevant for the respective targeted medical application.

Within the time frame of our experiments, we could not observe any alterations in the mechanical properties of THANOS. However, future studies have to determine its degradation rate and the demagnetization behaviour of THANOS. The latter depends on the properties of the ferromagnetic particles. According to the manufacturer's specification, MQFP™-16-7-11277 particles have a demagnetization factor of 0.21. Therefore, the particles have a relatively low susceptibility to demagnetization. Nevertheless, future investigations must study this aspect before transitioning to potential applications.

Furthermore, we expect that the ratio of ferromagnetic particles not only impacts the magnetic response of the corresponding soft robot, but also influences the mechanical properties of the polymeric matrix. Additionally, the size and composition of these particles are expected to play a significant role in determining the resulting properties. In this study, we focused on achieving complex locomotion of THANOS by using a straightforward synthesis method. Moving forward, further investigations into the optimal ratio, composition, and size of the ferromagnetic particles embedded in the polymeric matrix, as well as the monomer-to-crosslinker ratio, are necessary to obtain optimal properties for the desired application.

As mentioned in Section 3.3, the VPTT of the pNIPAM-based polymeric matrix can be tuned by altering its chemical composition. The current THANOS robot has a transition temperature of 37 °C. For the use in biomedical applications, future studies should customize the VPTT to align with temperatures closely resembling physiological conditions. Furthermore, alternative heating methods must be incorporated because heating through adjustments in the surrounding temperature would not be feasible. Consequently, either infrared irradiation or hyperthermia approaches would be necessary.^51^

Future studies need to verify the temperature response of THANOS using the above mentioned techniques. However, one can also envision the inclusion of other responses that can be more in line with biological environments such as stimulated bioadhesion.^52,53^ Besides tailoring the VPTT, future investigations should explore the integration of additional external stimuli, such as light or pH, within the polymeric matrix to ultimately broaden the applicability of these systems.^54^ The locomotion capabilities of the robot can be enhanced for applications in real body environments, such as biological tissues covered by a layer of mucus. Such a coating could also potentially enhance the robot's biocompatibility and prevent leakage of magnetic particles.^55^

## 5 Conclusions

This study introduces a dual-responsive thermo-magnetic soft robot, combining the advantages of temperature-responsive polymeric networks and magnetic actuation. The integration of ferromagnetic particles inside a pNIPAM matrix results in a versatile robot with programmable responses to both temperature changes and external magnetic fields. The synthesis method, employing one-step temperature-induced free radical polymerization with controlled cooling, ensures a straightforward fabrication process. The extremely short reaction time results in a homogeneous distribution of magnetic particles inside the polymeric network.

The presented soft robot exhibits a range of complex locomotion patterns, including rolling, undulating, gripping, and inchworm-like motion achieved through magnetic actuation. The shrinkage ability at elevated temperatures enables the robot to perform dual locomotion tasks for pick-and-place maneuvers of objects. First, the robot can be immobilized in a gripping state by magnetic actuation. Subsequently, the shape is locked under continuous magnetization by raising the temperature. Above the VPTT of pNIPAM, the polymer matrix transitions from a swollen to a collapsed state. This enables the robot to grab an object and maintain the desired shape autonomously, eliminating the necessity for continuous magnetic control. Secondly, the dual responsive behaviour allows the robot to reach the target location by magnetic field control. Ultimately, the object is released by decreasing the temperature, in which the robot transitions back to the initial swollen state.

Overall, this dual-responsive thermo-magnetic soft robot presents a promising advancement in the field of soft robotics, offering a versatile platform with intriguing possibilities for applications in biomedical domains, such as surgical procedures, where controlled and programmable responses to environmental stimuli are crucial.

## Conflicts of interest

The authors declare the following financial interests/personal relationships which may be considered as potential competing interests: Patrick van Rijn reports a relationship with BiomACS BV that includes equity or stocks. P.V.R is also the co-founder, scientific advisor, and share- holder of BiomACS BV, a biomedical oriented screening company. The authors declare no other competing interests. The authors declare no further conflict of interest.

## Supplementary Material

TB-012-D3TB02839A-s001

TB-012-D3TB02839A-s002
