# Supplementary material for: Minimally designed thermo-magnetic dual responsive soft robots for complex applications
Source: J Mater Chem B. 2024 Mar 28;12(22):5339–49. doi: 10.1039/d3tb02839a (PMC11152039; doi:10.1039/d3tb02839a)
Supplement: TB-012-D3TB02839A-s002 [file TB-012-D3TB02839A-s002.pdf]

Electronic Supplementary Material (ESI) for Journal of Materials Chemistry B.
This journal is © The Royal Society of Chemistry 2024

Supporting Information

Minimally designed thermo-magnetic dual responsive soft robots for complex applications

Clio Siebenmorgen*[a], Chen Wang *[a], Laurens Bosscher Navarro [a], Daniele Parisi [b], Sarthak Misra [ac], Venkatasubramanian Kalpathy Venkiteswaran[c‡] and Patrick van Rijn [a‡]

a University of Groningen, University Medical Center Groningen
Biomaterials & Biomedical Technology
A. Deusinglaan 1, 9713 AV Groningen, The Netherlands
b University of Groningen,
Faculty of Science and Engineering, Product Technology — Engineering and Technology Institute Groningen
Nijenborgh 4, 9747 AG Groningen, The Netherlands
c University of Twente
Surgical Robotics Laboratory, Department of Biomechanical Engineering
Drienerlolaan 5, 7522 NB Enschede, The Netherlands
* These authors contributed equally to this work
‡ Corresponding authors email: v.kalpathyvenkiteswaran@utwente.nl (V. Venkiteswaran) and p.van.rijn@umcg.nl (P. van Rijn)

Table of Contents

1. Section A: Supporting Notes

Note S1: Modeling

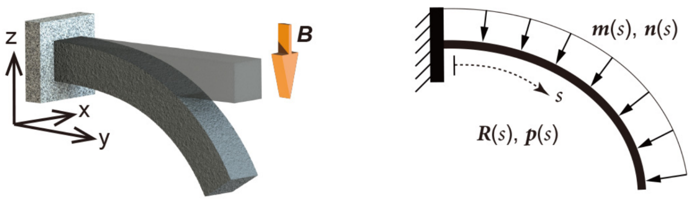

Figure S1: Schematic and illustration of the Cosserat rod theory. $\boldsymbol{R}(s), \boldsymbol{p}(s), \boldsymbol{n}(s)$ and $\boldsymbol{m}(s)$ are materials states vector at the position $s \in [0, l]$ along the rod. $\boldsymbol{B}$ indicates the magnetic field.

To assess the mechanical behavior of the robot subjected to pre-designed magnetization profiles under an external magnetic field, we utilize a model based on Cosserat rod theory. Additionally, this model aids in the robot's design process. In this model, the robot is conceptualized as a flexible rod (along the long axis of the robot), firmly attached at its proximal end to a rigid base while being free to move at its distal tip. The above figure provides a schematic illustration of how the robot bends under the influence of the magnetic field. This Cosserat rod model is coupled with magnetization profiles and magnetic fields, allowing us to simulate the deformation of the robots. Here, each rod is characterized by its centerline curve in three-dimensional space, denoted by $s \in [0, l]$, where $l \in \mathbb{R}^3$ represents the length of the robot. The discretized cross-section along s can be succinctly expressed by a material state vector:

$$\mathbf{y}(s) = [\boldsymbol{R}(s)\, \boldsymbol{p}(s)\, \boldsymbol{n}(s)\, \boldsymbol{q}(s)], \tag{1}$$

where $\boldsymbol{R}$ is rotation matrix of material orientation, $\boldsymbol{p}$ represents global position in Cartesian coordinates, $\boldsymbol{n}$ is internal force in the global frame, $\boldsymbol{q}$ is internal moment in the global frame.

The actuation magnetic field is generated using 3D electromagnetic coils (shown in Fig. S2) designed to produce a uniform magnetic field within the workspace, and therefore the contribution of the magnetic field gradient is assumed to be negligible. Consequently, the applied force distribution per unit of s is denoted as $\boldsymbol{f}(s) = 0$. Additionally, for the purposes of this study, gravity is disregarded. Therefore, the applied torque distribution per unit length of s can be represented as ($\boldsymbol{\tau} \in \mathbb{R}^3$), and is given by

$$\boldsymbol{\tau}(s) = \boldsymbol{m} \times \boldsymbol{B}, \tag{2}$$

where $\boldsymbol{B} \in \mathbb{R}^3$ is the magnetic flux density, $\boldsymbol{m} \in \mathbb{R}^3$ is the magnetic dipole moment per unit and is expressed as

$$\boldsymbol{m} = \frac{1}{\mu_0}\boldsymbol{B_r}\, V_{rob} = \iiint \boldsymbol{M_{rob}}\, dV_{rob}, \tag{3}$$

where μ_0 is the permeability of vacuum, $\boldsymbol{B_r}$ represents the residual flux density, V_{rob} is the volume of the robot, $\boldsymbol{M_{rob}}$ is the magnetization of the robot.

The equilibrium differential equations are expressed as:

$$\dot{\boldsymbol{n}}(s) + \boldsymbol{f}(s) = 0, \tag{4}$$

$$\dot{\boldsymbol{m}}(s) + \dot{\boldsymbol{p}}(s) \times \dot{\boldsymbol{n}}(s) + \boldsymbol{\tau}(s) = 0, \tag{5}$$

where the dot denotes a derivative with respect to s.

The boundary value problem of Cosserat rod is solved using the fourth-order Runge-Kutta method, implemented using Matlab (2021a, Mathworks, USA). The measured residual flux density and density of the magnetic particle used in this study is set to 911 mT and 7.61 g/cm^3 respectively. The detailed modeling and solution approach closely adhere to the methodologies outlined in [1, 2], and implementation detailed can be found in our previous work [3].

2. Section B: Supporting Figures

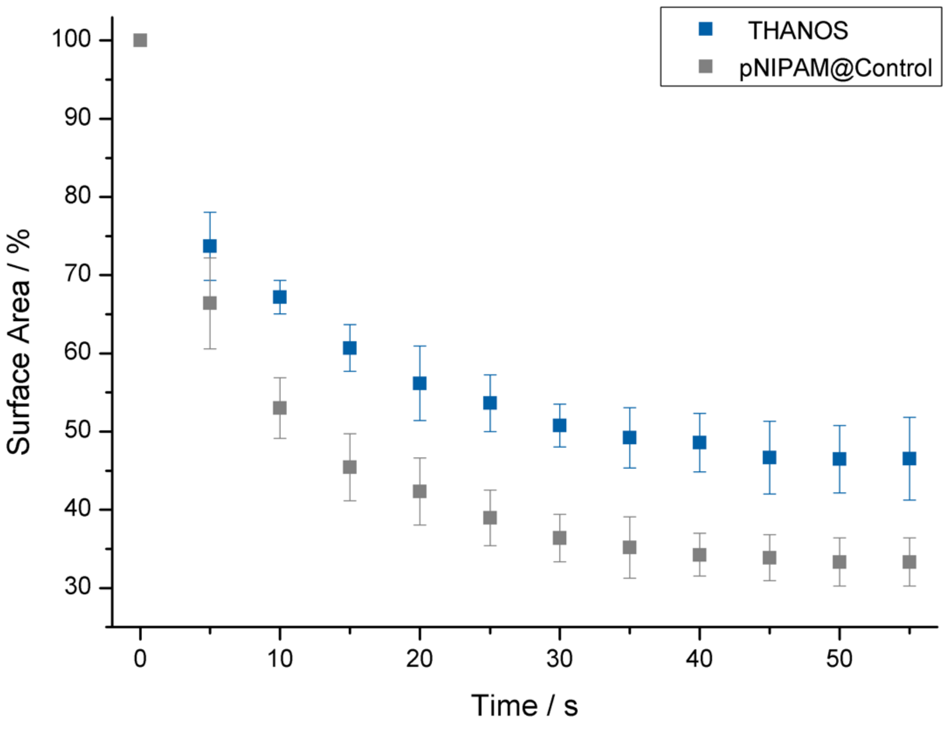

Figure S2: Characterization of the time dependent shrinking capability of THANOS (blue) and pNIPAM@Control (grey) at a temperature of 50°C.

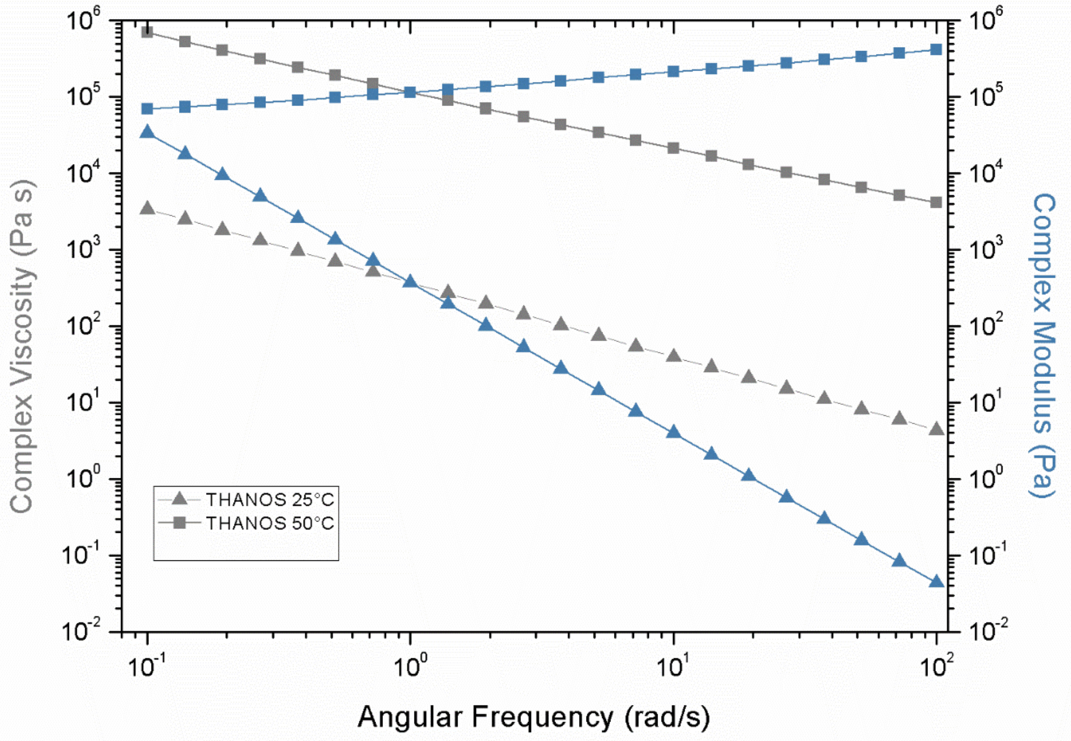

Figure S3: Complex Viscosity (grey) and Complex Modulus (blue) of THANOS depending on the Angular Frequency. Triangles show the values of THANOS at 25°C, while rectangles show the values of THANOS at 50°C.

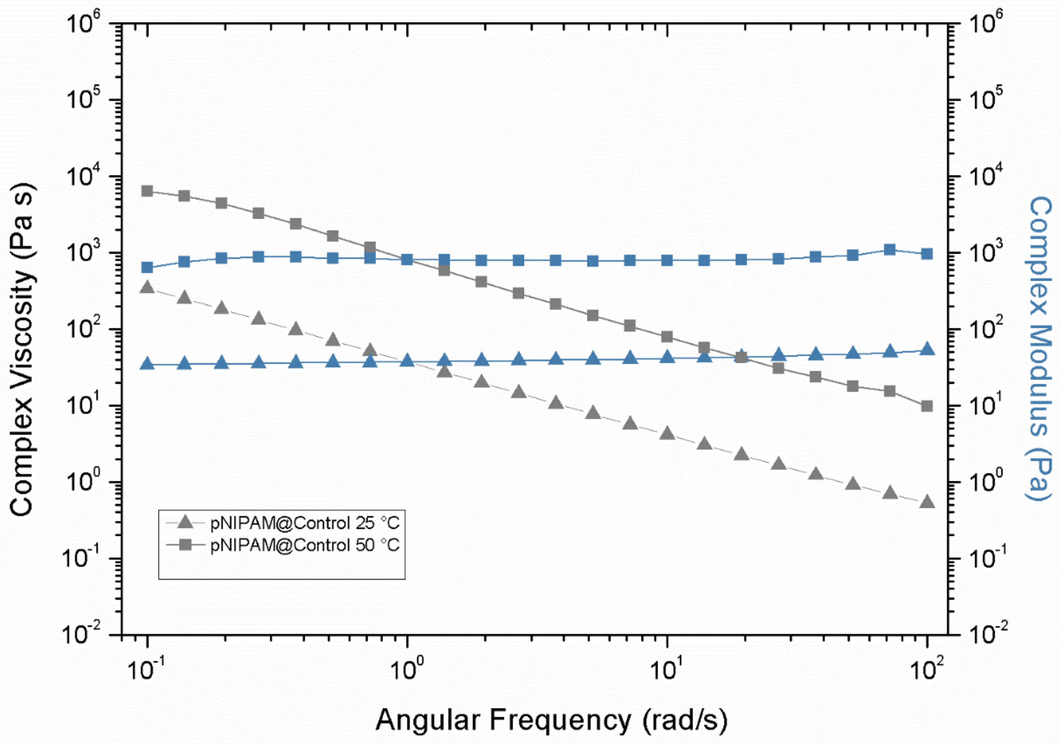

Figure S4: Complex Viscosity (grey) and Complex Modulus (blue) of pNIPAM@Control depending on the Angular Frequency. Triangles show the values of pNIPAM@Control at 25°C, while rectangles show the values of pNIPAM@Control at 50°C.

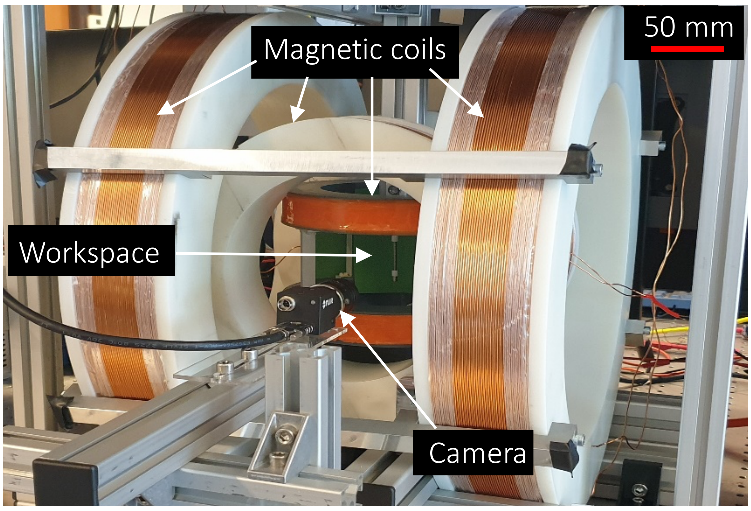

Figure S5: Magnetic actuation setup.

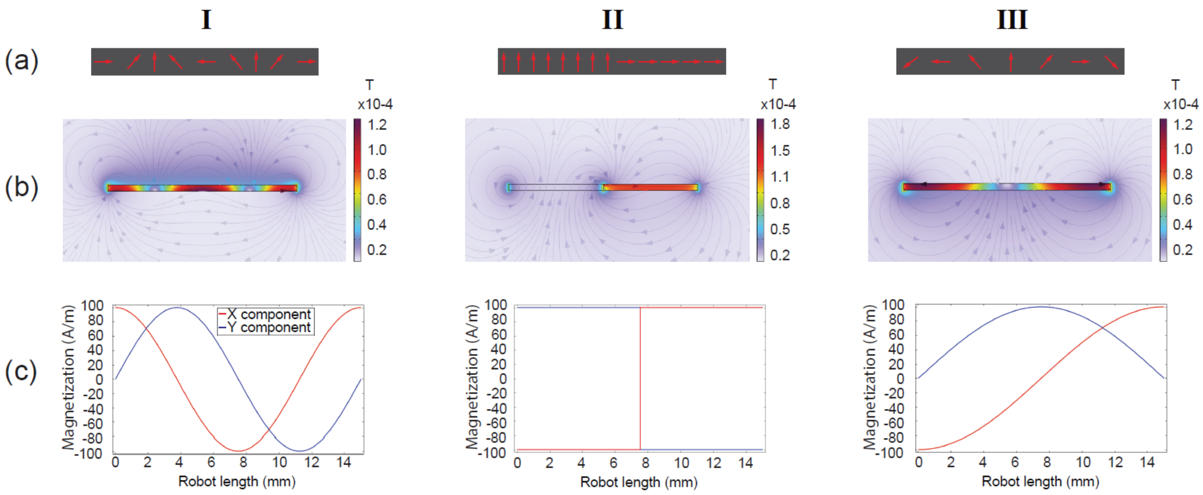

Figure S6: (a) Designed magnetization profile (shown in red arrows), (b) the magnetic flux density and (c) magnetization strength and direction (in X and Y component) of the **I** “S” **II** “L” and **III** “C” shaped robots.

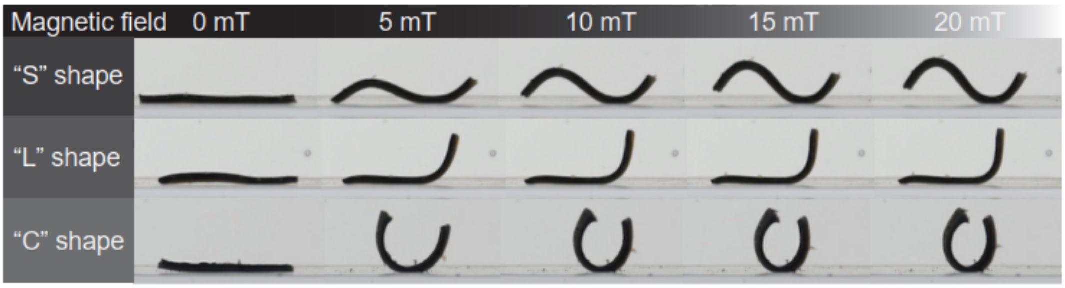

Figure S7: Snapshots of experiments with respect to the deformation of the “S”, “L” and “C” shaped robots under different magnetic field increased from 0 mT to 20 mT.

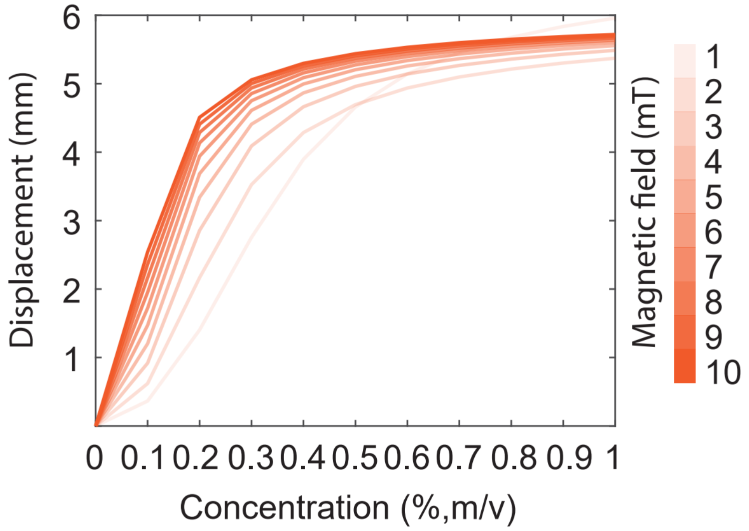

Figure S8: Simulation results of the influence of magnetic particle concentration on the displacement of the “L” shaped robot. The applied magnetic field is increased from 1 mT to 10 mT.

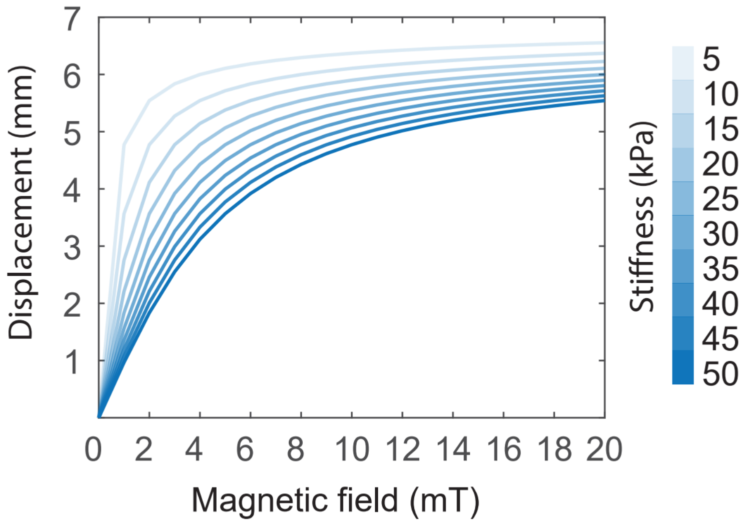

Figure S9: Simulation results of the influence of robot stiffness on the displacement of the “L” shaped robot. The applied magnetic field is increased from 1 mT to 20 mT.

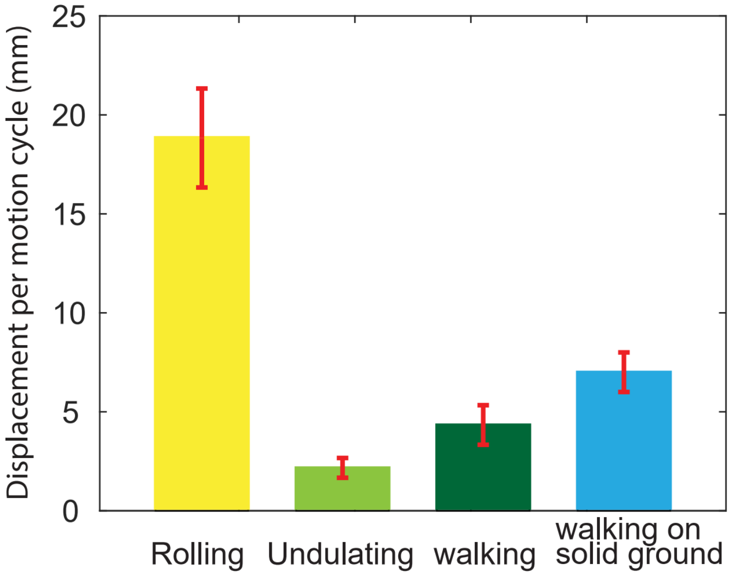

Figure S10: Experimental results: comparison of the displacement per motion cycle of the four motion patterns. Displacements are measured under different magnetic field strength and frequency.

References

[1] J. Till et al., “Real-time dynamics of soft and continuum robots based on cosserat rod models,” Int. J. Rob. Res., vol. 38, no. 6, pp. 723–746, 2019.

[2] D. Rucker and R. Webster III, “Statics and dynamics of continuum robots with general tendon routing and external loading,” IEEE Trans. Robot., vol. 27, no. 6, pp. 1033–1044, 2011.

[3] M. Richter et al., “Multi-point orientation control of discretelymagnetized continuum manipulators,” IEEE Robot. Autom. Lett., vol. 6, no. 2, pp. 3607–3614, 2021.
